# Optimization and in vitro antiproliferation of *Curcuma wenyujin*’s active extracts by ultrasonication and response surface methodology

**DOI:** 10.1186/s13065-016-0177-9

**Published:** 2016-05-16

**Authors:** Xiaoqin Wang, Ying Jiang, Daode Hu

**Affiliations:** Department of Clinical Pharmacology, Shanghai General Hospital, Shanghai Jiao Tong University School of Medicine, 100 Haining Road, Shanghai, 200080 China

**Keywords:** Ultrasonic extraction, Response surface methodology, *Curcuma wenyujin*, High performance liquid chromatography, Antiproliferative activity

## Abstract

**Background:**

*Curcuma wenyujin*, a member of the genus *Curcuma*, has been widely prescribed for anti-cancer therapy. Multiple response surface optimization has attracted a great attention, while, the research about optimizing three or more responses employing response surface methodology (RSM) was very few.

**Results:**

RSM and desirability function (DF) were employed to get the optimum ultrasonic extraction parameters, in which the extraction yields of curdione, furanodienone, curcumol and germacrone from *C. wenyujin* were maximum. The yields in the extract were accurately quantified using the validated high performance liquid chromatography method with a good precision and accuracy. The optimization results indicated that the maximum combined desirability 97.1 % was achieved at conditions as follows: liquid–solid ratio, 8 mL g^−1^; ethanol concentration, 70 % and ultrasonic time, 20 min. The extraction yields gained from three verification experiments were in fine agreement with those of the model’s predictions. The surface morphologies of the sonication-treated *C. wenyujin* were loose and rough. The extract of *C. wenyujin* presented obvious antiproliferative activities against RKO and HT-29 cells in vitro.

**Conclusion:**

Response surface methodology was successfully applied to model and optimize the ultrasonic extraction of four bioactive components from *C. wenyujin* for antiproliferative activitiy.

**Graphical abstract:**
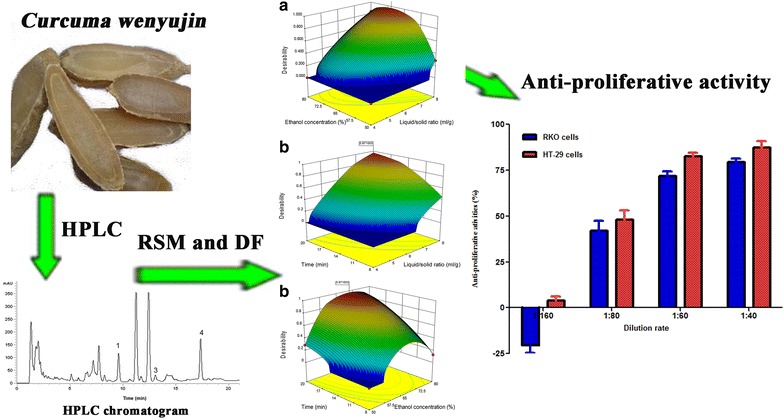
.

**Electronic supplementary material:**

The online version of this article (doi:10.1186/s13065-016-0177-9) contains supplementary material, which is available to authorized users.

## Background

*Rhizoma Curcumae*, a number of the genus *Curcuma*, is cultivated in tropical and subtropical countries [1]. In Chinese Pharmacopoeia, *R. Curcumae* means the rhizomes derived from *Curcuma phaeocauli*s Val., *C. kwangsiensis* S.G. Lee et C.F. Liang or *C. wenyujin* Y.H. Chen et C. Ling [2, 3]. Recently, it is broadly prescribed as an anti-cancer drug in some Asian countries, such as China [4, 5]. Sesquiterpenes, the main biological active compotents in *R. Curcumae*, such as germacrone, curcumol and furanodienone, possess powerful anti-cancer properties against breast cancer, liver cancer and lung cancer [4–8]. Moreover, curcumol, germacrone and curdione have been chosen as the index ingredients for its quality control [9, 10]. As for the quantitative analysis of these volatile components with thermo-sensitive and biological ability in *R. Curcumae*, high performance liquid chromatography (HPLC) is more suitable than gas chromatography-mass spectrometry [3].

Currently, ultrasonic extraction and supercritical fluid extraction (SFE) are gradually substituting the conventional extraction methods [11–13]. However, the system for SFE is a bit complicated and expensive [14]. Ultrasonic extraction can achieve a high extraction efficiency in a very short period of time through promoting the liquids with different poralities to generate fine emulsions and accelerating the mass-transfer procedure in the reaction system [15–17]. For these reasons, ultrasonic extraction has been broadly adopted in extraction with advantages of saving time [18] and protecting heat-sensitive bioactive compounds from damage at a lower performance temperature [19].

Many parameters, such as ultrasonic time and solvent composition can influence the ultrasonic extraction efficiency separately or jointly [20]. With the aid of central composite design (CCD), response surface methodology (RSM) has been a very useful tool to investigate the individual or collective effects of several parameters on responses [20]. Further, desirability function (DF) can optimize performance conditions for one or more responses simultaneously via combining several responses into one [17]. Now, the RSM coupled with DF has been employed to optimize extraction process [20] and prepare nanoparticles [21]. However, the research about optimizing on three or more responses via employing RSM and DF was very few.

Due to the complexity of the compotents in herbs, combined action often occurs, bringing in an improvement of the therapeutic effect [9]. Currently, a great attention has been given to the biological activities of Chinese medical herb extracts and its mechanisms [22–24].

This study focused on optimizing the ultrasonic extraction conditions to achieve the maximum extraction yields of four bioactive compotents from *C. wenyujin* by employing RSM coupled with DF and evaluating the antiproliferative activities of the *C. wenyujin* extract against two colorectal cancer (CRC) cell lines. Meanwhile, the impacts of ultrasound on the surface morphologies of *C. wenyujin* were explored.

## Results and discussion

### Analytical performance of high performance liquid chromatography

**Fig. 1:**
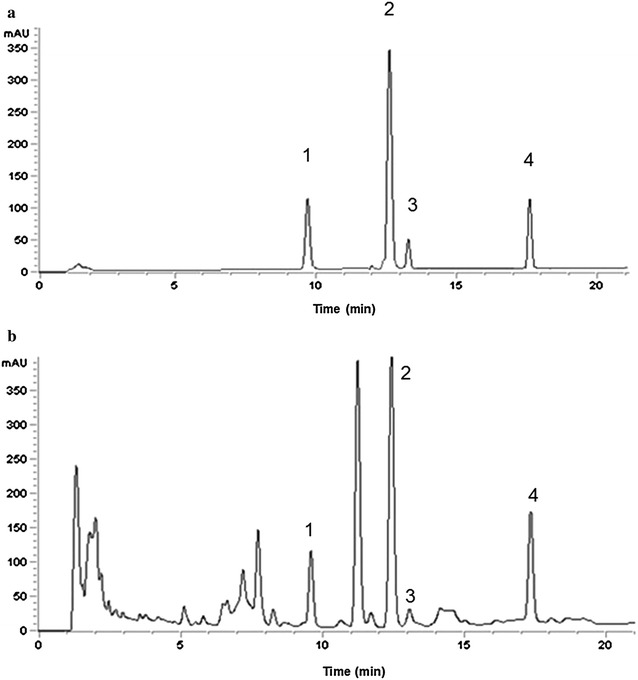
HPLC chromatograms of **a** mixed standards of the four volatile components and **b** the four components in *Curcuma wenyujin*: (1) curdione; (2) furanodienone; (3) curcumol and (4) germacrone

The HPLC prolife of the extract of *C. wenyujin* was demonstrated in Fig. 1. As expected, four peaks indicated curdione, furanodienone, curcumol and germacrone were identified, respectively. The HPLC method was validated through studying the regression equations, limits of detection (LOD) and so on, as displayed in Additional file 1: Table S1. The precision of the method was examined by analyzing the intra- and inter-day variations. The relative standard deviations (RSDs) for the intra-day variabilities of the four tested compounds were 1.57, 1.77, 4.18 and 2.04 %, respectively, and the RSDs for the inter-day variabilities were 1.13, 0.56, 5.61 and 1.47 %, respectively, indicating a high accuracy. The recoveries for the four compotents were in the range of 97.91–104.28 % with RSD ranging from 3.69 to 4.82 %. Summarily, the validated HPLC method was suitable for quantifing the yields of these four bioactive compotents in the extract of *C. wenyujin*.

### Single factor tests

Single factor tests were adopted to evaluate whether the type of solvent, solvent concentration, liquid–solid ratio, ultrasonic time and extraction temperature could be optimized for ultrasonic extraction yields of these four bioactive compotents from *C. wenyujin*, and the results are displayed in Additional file 2: Figure S1.

Additional file 2: Figure S1a demonstrates that the extraction potential of ethanol is the second strongest, which is weaker than that of methanol, but stronger than those of ether and ethyl acetate. Besides, ethanol is safe and eco-friendly compared with methanol. Especially, Chen et al. adopted ethanol to prepare *C. phaeocaulis* Val. extract with anti-tumor potential [24]. Therefore, ethanol was chosen as solvent for next single factor tests.

Additional file 2: Figure S1b displays that the total extraction yield started to increase with increasing ethanol concentration, and peaked to the maximal yield 3.85 mg g^−1^ at concentration 80 % and then decreased, consistent to Xu’s result [20]. Taking the extraction yield and solvent consumption into consideration, 70 % was selected as the solvent concentration for next analysis.

Additional file 2: Figure S1c reveals that the total extraction yield was positively and significantly increased by the liquid–solid ratio until the ratio was beyond 8 mL g^−1^; after that, the yield was almost unchanged. Generally speaking, a higher solvent ratio can dissolve components more effectively from herbal materials, bringing in a promoted extraction efficiency [25]. Whereas excessive solvent will cause extra workload in the concentration process [25]. Therefore, 8 mL g^−1^ was ascertained as the liquid–solid ratio.

Additional file 2: Figure S1d presents that the extraction yield increased as the ultrasonic time increased from 3 to 15 min. An adequate extraction time would be beneficial for promoting the extraction efficiency, while inordinately long extraction time might cause loss of activities [20]. Accordingly, we fixed the ultrasonic time at 15 min.

As we can see, the extraction yield was almost unchanged when the extraction temperature changed from 20 to 50 °C (Additional file 2: Figure S1e). Besides, a higher extraction temperature probably was not good for thermo-sensitive bioactive compotents, such as germacrone in *R. Curcumae*, leading to loss of activities [3, 20]. Thus, the extraction temperature was set at 30 °C for further optimization experiments.

Three factors, the ethanol concentration, liquid–solid ratio and ultrasonic time, were chosen for further optimizing ultrasonic extraction conditions of the four bioactive compotents from *C. wenyujin* by the subsequent RSM coupled with DF.

### Optimization employing response surface methodology

#### Statistical analysis and the model fitting

The data about the opration conditions of 17 runs and the four responses are presented in Table 1. The analysis of variance (ANOVA) was employed to verify the correctness of the quadratic models, as presented in Table 2. The contributions of the models for these four compotents were significant for the *p* values were less than 0.05. The regression coefficients of the coded models for these four compounds are given in Table 2. Similarly, liquid–solid ratio (*X*_*1*_), ethanol concentration (*X*_*2*_), ultrasonic time (*X*_*3*_) and quadratic ethanol concentration (*X*_*2*_^*2*^) are significant model terms. Moreover, the contributions of the three significant variables on the yields of the four compotents could be ranked in the following orders: ultrasonic time (*X*_*3*_) < ethanol concentration (*X*_*2*_) < liquid–solid ratio (*X*_*1*_). The lack of fit were not statistically significant (*p* = 0.4281, 0.4963, 0.2232 and 0.1346, Table 2), suggesting the models fitted the data well.

**Table 1:** Central composite design and results for ultrasonic extraction of curdione, furanodienone, curcumol and germacrone from *Curcuma wenyujin*

Run	Factors			Curdione (mg g^−1^)	Furanodienone (mg g^−1^)	Curcumol (mg g^−1^)	Germacrone (mg g^−1^)	Total yield (mg g^−1^)
	***X***_***1***_	***X***_***2***_	***X***_***3***_					
1	2.6	6.5	14	1.53	0.97	0.16	0.24	2.90
2	6	65	14	1.77	1.32	0.21	0.34	3.64
3	4	50	20	1.34	0.90	0.14	0.23	2.61
4	6	65	3.9	1.62	1.04	0.16	0.26	3.08
5	4	80	20	1.73	1.19	0.20	0.31	3.43
6	6	65	24.1	1.84	1.45	0.22	0.38	3.89
7	8	80	20	2.00	1.50	0.25	0.38	4.13
8	4	80	8	1.57	1.18	0.18	0.29	3.22
9	6	39.8	14	1.38	0.74	0.12	0.18	2.42
10	6	65	14	1.87	1.43	0.20	0.36	3.86
11	6	65	14	1.75	1.39	0.22	0.35	3.71
12	9.4	65	14	1.92	1.47	0.25	0.39	4.03
13	8	50	20	1.80	1.29	0.20	0.33	3.62
14	8	80	8	1.83	1.30	0.21	0.33	3.67
15	4	50	8	1.25	0.78	0.12	0.20	2.35
16	6	90.2	14	1.60	1.11	0.16	0.24	3.11
17	8	50	8	1.82	1.17	0.18	0.30	3.47

*X*_*1*_ Liquid to solid ratio (mL g^−1^); *X*_*2*_ Ethanol concentration (%); *X*_*3*_ Ultrasonic time (min)

**Table 2:** Analysis of variance for central composite design and tests of the regression coefficients and intercepts of coded equations for curdione, furanodienone, curcumol and germacrone

Source		Mean squares	F	*p* value	Coefficient estimate
Curdione	Model	0.075	12.99	0.0014	
	Intercept				1.790
	*X*_*1*_	0.360	61.88	0.0001	0.160
	*X*_*2*_	0.120	20.88	0.0026	0.094
	*X*_*3*_	0.042	7.29	0.0307	0.056
	*X*_*1*_ *X*_*2*_	0.029	5.04	0.0596	−0.060
	*X*_*1*_ *X*_*3*_	1.326 × 10^−3^	0.23	0.6466	−0.013
	*X*_*2*_ *X*_*3*_	8.001 × 10^−3^	1.38	0.2779	0.032
	*X*_*1*_^*2*^	4.588 × 10^−3^	0.79	0.4027	−0.020
	*X*_*2*_^*2*^	0.110	19.80	0.0030	−0.100
	*X*_*3*_^*2*^	3.531 × 10^−3^	0.61	0.4601	−0.018
	Lack of Fit	6.474 × 10^−3^	1.60	0.4281	
	*R* ^*2*^ = 0.9435, *Q* ^*2*^ = 0.8677, Adeq Precision = 12.121				
Furanodienone	Model	0.098	27.14	0.0001	
	Intercept				1.370
	*X*_*1*_	0.330	90.16	<0.0001	0.150
	*X*_*2*_	0.190	51.54	0.0002	0.120
	*X*_*3*_	0.110	29.00	0.0010	0.088
	*X*_*1*_ *X*_*2*_	9.730 × 10^−3^	2.69	0.1452	−0.035
	*X*_*1*_ *X*_*3*_	1.966 × 10^−3^	0.54	0.4852	−0.013
	*X*_*2*_ *X*_*3*_	1.566 × 10^−4^	0.04	0.8412	4.425 × 10^−3^
	*X*_*1*_^*2*^	0.025	7.01	0.0331	−0.047
	*X*_*2*_^*2*^	0.250	70.00	<0.0001	−0.150
	*X*_*3*_^*2*^	0.015	4.14	0.0813	−0.036
	Lack of Fit	3.853 × 10^−3^	1.27	0.4963	
	*R* ^*2*^ = 0.9721, *Q* ^*2*^ = 0.9117, Adeq Precision = 16.176				
Curcumol	Model	2.694 × 10^−3^	15.43	0.0008	
	Intercept				0.210
	*X*_*1*_	8.931 × 10^−3^	51.15	0.0002	0.026
	*X*_*2*_	4.953 × 10^−3^	28.37	0.0011	0.019
	*X*_*3*_	2.519 × 10^−3^	14.43	0.0067	0.014
	*X*_*1*_ *X*_*2*_	1.739 × 10^−4^	1.00	0.3515	−4.663 × 10^−3^
	*X*_*1*_ *X*_*3*_	9.453 × 10^−5^	0.54	0.4858	3.437 × 10^−3^
	*X*_*2*_ *X*_*3*_	9.045 × 10^−5^	0.52	0.4950	3.362 × 10^−3^
	*X*_*1*_^*2*^	3.674 × 10^−7^	2.10 × 10^−3^	0.9647	−1.805 × 10^−4^
	*X*_*2*_^*2*^	6.848 × 10^−3^	39.22	0.0004	−0.025
	*X*_*3*_^*2*^	2.553 × 10^−4^	1.46	0.2658	−4.759 × 10^−3^
	Lack of Fit	2.209 × 10^−4^	3.76	0.2232	
	*R* ^*2*^ = 0.9520, *Q* ^*2*^ = 0.8957, Adeq Precision = 14.233				
Germacrone	Model	7.756 × 10^−3^	16.36	0.0007	
	Intercept				0.350
	*X*_*1*_	0.023	49.39	0.0002	0.041
	*X*_*2*_	0.010	21.15	0.0025	0.027
	*X*_*3*_	9.435 × 10^−3^	19.90	0.0029	0.026
	*X*_*1*_ *X*_*2*_	8.694 × 10^−4^	1.83	0.2178	−0.010
	*X*_*1*_ *X*_*3*_	9.940 × 10^−5^	0.21	0.6609	3.525 × 10^−3^
	*X*_*2*_ *X*_*3*_	1.682 × 10^−5^	0.04	0.8559	1.450 × 10^−3^
	*X*_*1*_^*2*^	7.945 × 10^−4^	1.68	0.2365	−8.395 × 10^−3^
	*X*_*2*_^*2*^	0.025	52.89	0.0002	−0.047
	*X*_*3*_^*2*^	4.873 × 10^−4^	1.03	0.3444	−6.574 × 10^−3^
	Lack of Fit	6.264 × 10^−4^	6.72	0.1346	
	*R* ^*2*^ = 0.9546, *Q* ^*2*^ = 0.9076, Adeq Precision = 13.465				

*X*_*1*_ Liquid to solid ratio (mL g^−1^); *X*_*2*_ Ethanol concentration (%); *X*_*3*_ Ultrasonic time (min)

The determination coefficient (*R*^*2*^) is another index of model quality. For example, the determination coefficient for the model of curdione (*R*^*2*^ = 0.9435) suggested that 94.35 % of the variation for the curdione yield would be interpreted by the model [26]. As shown in Table 2, the determination coefficients of these four models ranged from 0.9435 to 0.9721, impling good fits between the actual data and the empirical models. It is obvious that the test objects were uniformly distributed and covered the whole range of the training set, as indicated in Additional file 3: Figure S2. Besides, the predictive squared correlation coefficients (*Q*^*2*^) [27] of these four models were 0.8677, 0.9117, 0.8957 and 9076, as displayed in Table 2. Therefore, each model possesses a high predictive ability [27]. The comparison of several methods often encounters problems, such as not very fair, which could be avoided by the sum of ranking differences (SRD) [28]. Therefore, we also employed SRD to evaluate the goodness of fit between the actual and the predicted value for these four models by a software named SRDrep (SRD with ties) [28, 29]. In the present study, the SRD values were 23, 14, 17 and 10 for the models of curdione, furanodienone, curcumol and germacrone, respectively, suggesting insignificant difference (*p* < 0.05) between the actual and the predicted value for these four models.

From the above statistical results, it is possible to regress the following second order polynomial equations:

1 $$ Y_{\text{curdione}} = - 1. 9 5 4 { } + \, 0. 2 8 7X_{1} + \, 0.0 7 2X_{2} + { 6}. 6 20 \, \times { 1}0^{ - 3} X_{3} - 4. 4 7 9 { } \times { 1}0^{ - 3} X_{2}^{2} $$

2 $$ Y_{\text{furanodienone}} = - 3. 5 4 1 { } + \, 0. 2 7 7X_{1} + \, 0. 10 1X_{2} + \, 0.0 3 2X_{3} - 0.0 1 2X_{1}^{2} - 6. 6 6 4 { } \times { 1}0^{ - 4} X_{2}^{2} $$

3 $$ Y_{\text{curcumol}} = - 0. 4 7 2 { } + \, 0.0 20X_{1} + \, 0.0 1 6X_{2} + { 1}. 8 1 8 { } \times { 1}0^{ - 3} X_{3} - 1.0 9 5 { } \times { 1}0^{ - 4} X_{2}^{2} $$

4 $$ Y_{\text{germacrone}} = - 1.0 4 5 { } + \, 0.0 6 4X_{1} + \, 0.0 3 1X_{2} + { 6}. 6 8 4 { } \times { 1}0^{ - 3} X_{3} - 2.0 9 6 { } \times { 1}0^{ - 4} X_{2}^{2} . $$

#### Response surface analysis

**Fig. 2:**
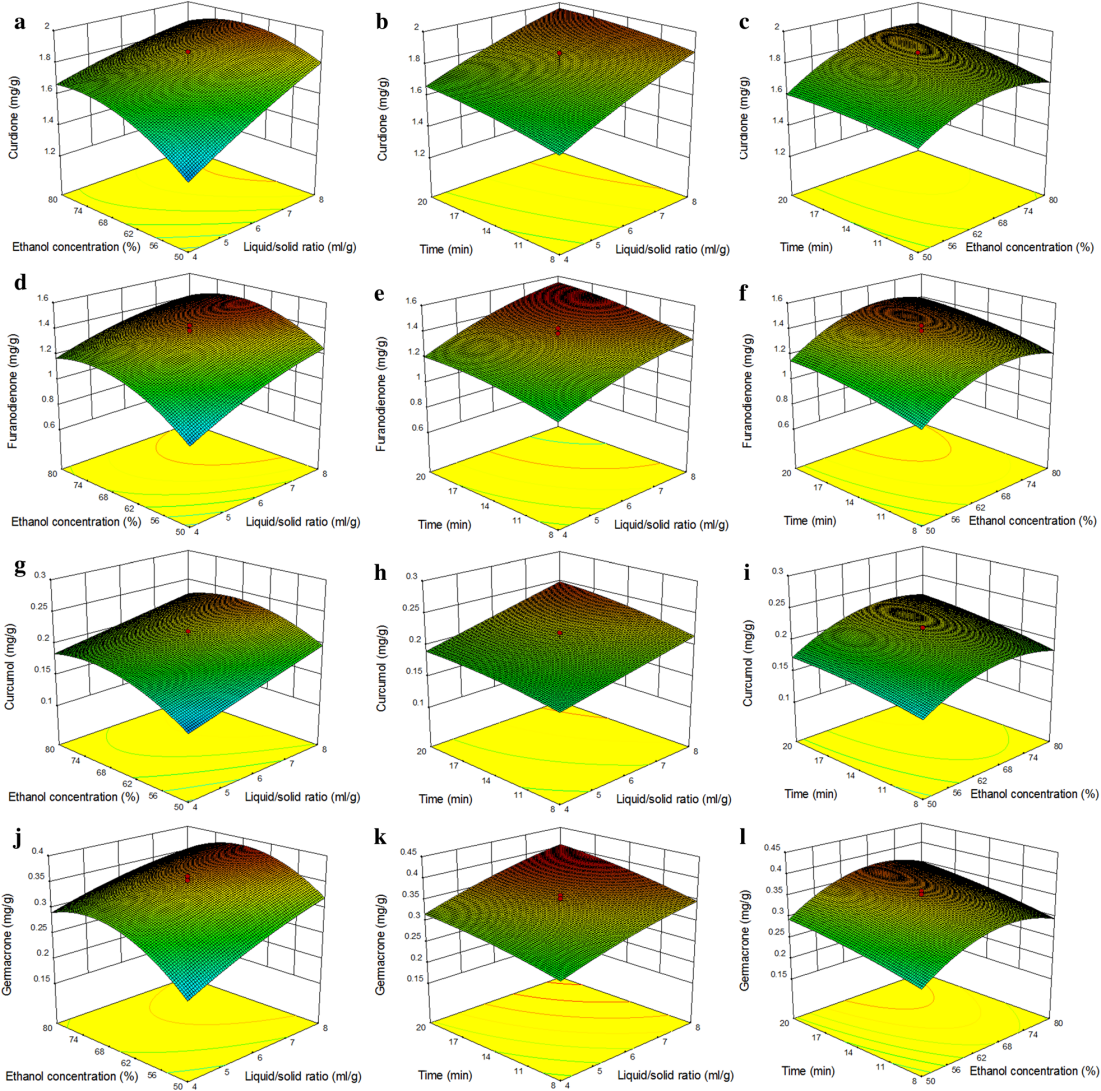
Three-dimensional response surface plots showing the effects of experimental factors and their mutual functions on extraction of: **a**–**c** Curdione; **d**–**f** Furanodienone; **g**–**i** Curcumol and **j**–**l** Germacrone from *Curcuma wenyujin*. The unmarked factor in each *plot* is held at its central value

Three-dimensional response surface plots were depicted to study the individual or collective effects of these three vital parameters on the ultrasonic extraction yields of these four main compotents from *C. wenyujin* (Fig. 2).

Figure 2a, d, g and j reveal that the interactive effects of liquid–solid ratio (*X*_*1*_) and ethanol concentration (*X*_*2*_) on the yields of the four compotents in 14 min of ultrasonic time (*X*_*3*_). Although the interaction are not statistically significant (*p* > 0.05, Table 2), the variation of these four compotent yields in the extracts can also be seen in these figures. When the two factors were at high levels, the extraction yields were maximum. At a given ethanol concentration, the yields increased as the liquid–solid ratio increased. While, the increment of the liquid–solid ratio failed to enhance the extraction yields obviously with the ratio in the range 7–8 mL g^−1^. This outcome was corresponding to the principle of mass transfer, where the transport force stems from the concentration gradient of a particular component between the solid and the liquid [26]. The transport force increases when a higher liquid–solid ratio is used [26]. However, the driving force will not increase when the solvent volume is sufficient [26]. In our study, the extraction yields were not significantly changed when the ratio was over 7 mL g^−1^, in agreement with the reports by Tian and Lou [26, 30].

Figure 2c, f, i and l indicate the insignificant functions of ethanol concentration (*X*_*2*_) and ultrasonic time (*X*_*3*_) for the extraction yields of these four compotents (*p* > 0.05, Table 2). As shown, the extraction yields were positively correlated with ethanol concentration when it was lower than about 70 %. However, they were negatively correlated when ethanol concentration increased beyond about 70 %, consistent with the quadratic coefficients of ethanol concentration (−0.100, −0.150, −0.025 and −0.047, respectively, Table 2). Previous studies reported that the ethanol solution with concentration ranging from 70 to 80 % (v/v) was suitable for extracting lipophilic phytochemicals, such as isorhamnetin and piceatannol [20, 31]. In aqueous organic solution, the dried herbal materials in dehydrated state could swell. Besides, according to the ‘‘like dissolves like’’ extraction principle, extracting lipophilic compotents should use organic solvents [31]. So, the action of ethanol concentration on extraction yield results from its function on expanding the herbs and promoting the dissolution of sesquiterpene compotents from the herbs [31].

Figure 2b, e, h and k present that the mutual influences of liquid–solid ratio (*X*_*1*_) and ultrasonic time (*X*_*3*_) were not correlated with the ultrasonic extraction yields of these four compotents (*p* > 0.05, Table 2). Fixing the liquid–solid ratio at 6 mL g^−1^, the extraction yields increased with ultrasonic time between 8 and 20 min, indicating the positive influence of ultrasonic time on the ultrasonic extraction efficiency. While, the increase in extraction yields was not particularly evident, when the ultrasonic time was above 17 min. Obviously, when the ethanol concentration was set at 65 %, the highest extraction yields could be gained at the ultrasonic time of 20 min and liquid–solid ratio of 8 mL g^−1^. Our result was similar to that of Wang et al. suggested that after the highest extraction yield was obtained, a extended ultrasonic time was not necessary [32].

The response surface plots indicated that the extraction yields mainly depended on the liquid–solid ratio, ethanol concentration and ultrasonic time, whereas no significant impact was observed in the mutual functions of these vital parameters, in good agreement with the ANOVA results.

#### Optimization using desirability function

**Fig. 3:**
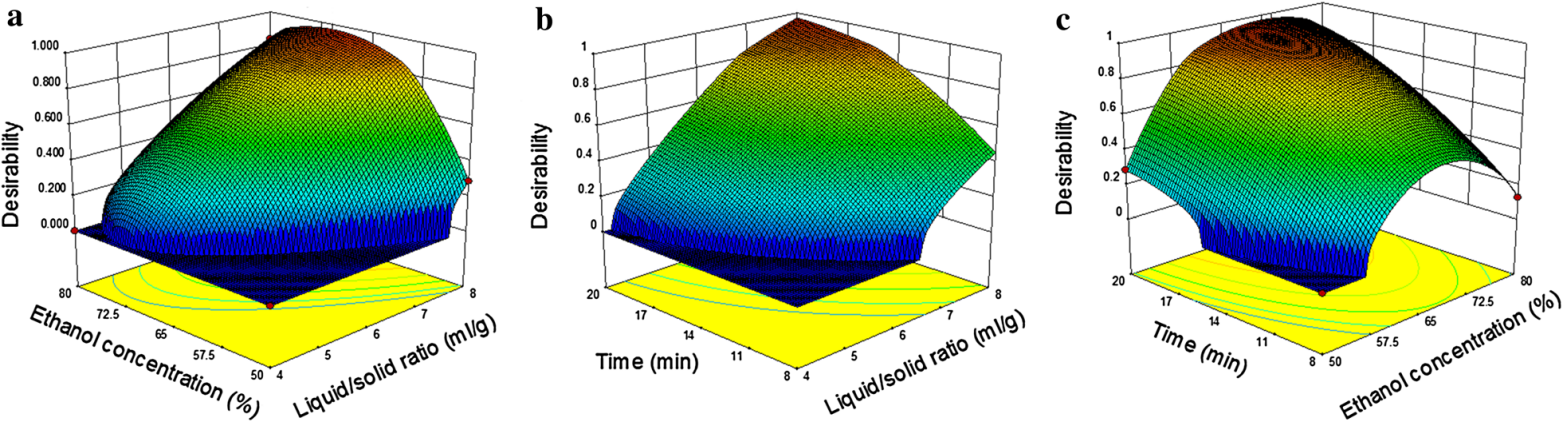
Response surface graph of the maximum global desirability function with 0.971 at **a** 20 min extraction time; **b** 70.1 % ethanol concentration and **c** 8 mL g^−1^ liquid–solid ratio

Based on the results of CCD, a DF approach was performed to achieve the purpose of optimizing the four responses continuously. The response surfaces of the combined desirability (*D*) were obtained, as illustrated in Fig. 3, bringing in the maximum *D* at the top with a condition as follows: liquid–solid ratio, 8 mL g^−1^; ethanol concentration, 70 % and ultrasonic time, 20 min. The maximum yields predicted for the four compotents were 1.97, 1.56, 0.25 and 0.41 mg g^−1^, respectively. Additional file 4: Figure S3 illustrates that the desirabilities of these four compounds were more than 0.9. Furthermore, the maximum *D* 0.971 was calculated out on the principle of *D* (*D* = *d*_*1*_ × *d*_*2*_ × *d*_*3*_ × *d*_*4*_ = 0.905 × 1 × 0.983 × 1 = 0.971). The optimization result was considered as acceptable and excellent with desirability value ranging from 0.8 to 1 [33]. In summary, the multiple response surface optimization result of this study was desirable.

#### Verification

Three verification experiments were performed to validate the ultrasonic extraction conditions optimized. Mean extraction yields of curdione, furanodienone, curcumol and germacrone were 1.98, 1.55, 0.25 and 0.40 mg g^−1^, respectively, consistent with the model’s predictions. Therefore, the ultrasonic extraction conditions for extracting the four bioactive compotents from *C. wenyujin* could be effectively optimized by employing RSM and DF.

### Comparison and field emission scanning electron micrographs

The optimizated ultrasonic extraction method was compared with the steam distillation (SD) extraction and maceration extraction. The results are presented in Table 3. The ANOVA results indicated that the total extraction yield of these four compotents gained by ultrasonic extraction was the highest at 4.19 mg g^−1^, followed by those of SD extraction and maceration extraction, with extraction time of 20 min (*p* < 0.05). Besides, SD extraction and maceration extraction took 1 and 2 h, respectively, to gain the similar extraction yields of the four compounds to that gained under the optimized ultrasonic extraction conditions. Combined with prior literature [34], our ultrasonic extraction method reduced the extraction time obviously.

**Table 3:** Extraction yields of curdione, furanodienone, curcumol and germacrone from *Curcuma wenyujin* by ultrasonic extraction, SD extraction and maceration extraction

Extraction methods	Extraction solvents	Extraction time	Curdione (mg g^−1^)	Furanodienone (mg g^−1^)	Curcumol (mg g^−1^)	Germacrone (mg g^−1^)	Total yield (mg g^−1^)
Ultrasonic extraction	70 % ethanol	20 min	2.00	1.56	0.25	0.41	4.22
SD extraction	Pure water	20 min	1.38	1.42	0.22	0.32	3.34
Maceration extraction	70 % ethanol	20 min	1.46	1.12	0.20	0.27	3.05
SD extraction	Pure water	1 h	1.89	1.52	0.24	0.39	4.04
Maceration extraction	70 % ethanol	2 h	1.94	1.52	0.26	0.38	4.10

SD means steam distillation

**Fig. 4:**
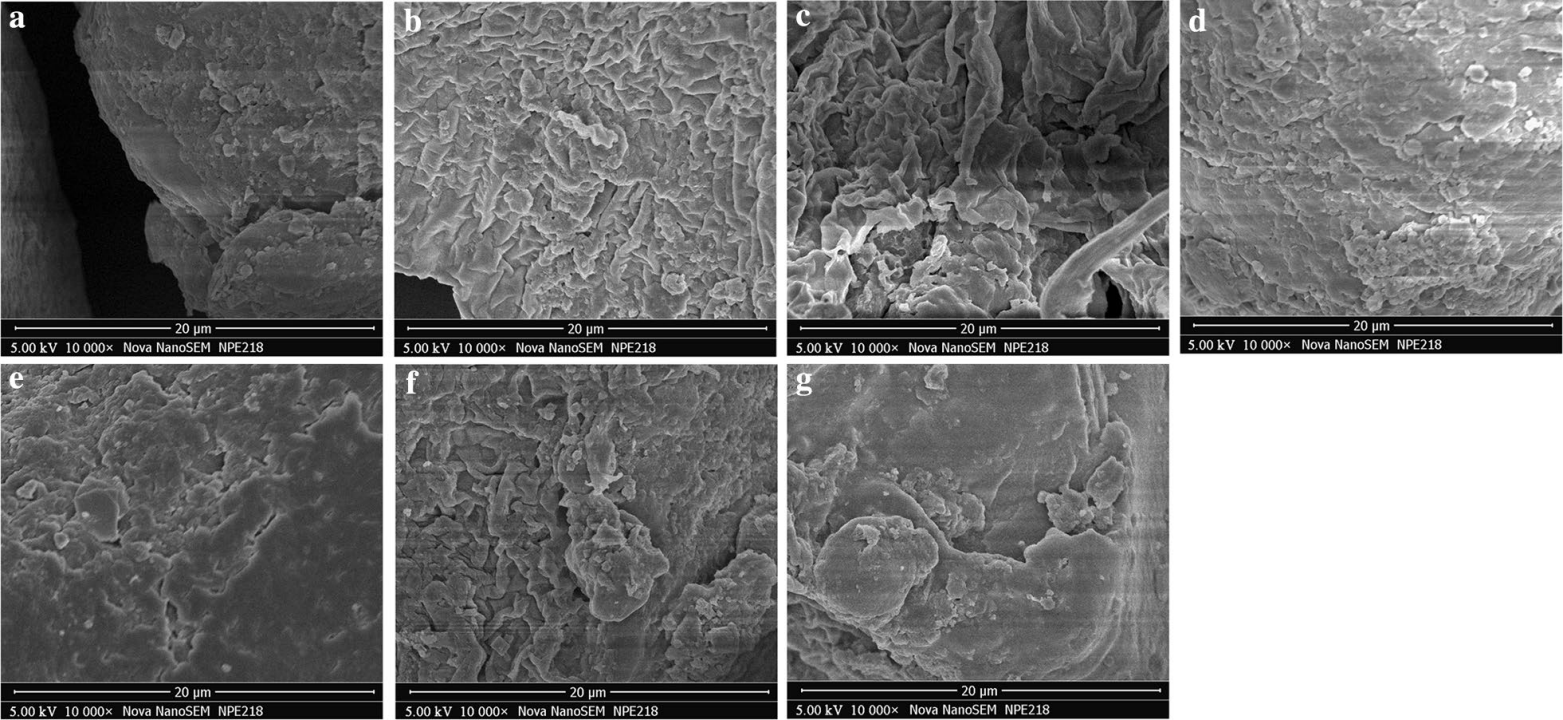
FESEM images of raw and treated materials under different extraction conditions. **a** Raw materials; **b** Ultrasonic extraction 8 min; **c** Ultrasonic extraction 20 min; **d** SD extraction 20 min; **e** Maceration extraction 20 min; **f** SD extraction 1 h and **g** Maceration extraction 2 h

For elucidating the mechanism of ultrasonic extraction, the characterization of *C. wenyujin* samples from ultrasonic extraction, SD extraction and maceration extraction were examined by field emission scanning electron microscope (FESEM, JEOL Ltd., Japan; Fig. 4). Comparing to the tight and smooth surface morphologies of raw *C. wenyujin* samples in Fig. 4a, we can see that the surface morphologies of ultrasound-treated *C. wenyujin* samples became loose and rough. Besides, a longer ultrasonic extraction time brought serious changes in surface morphology (Fig. 4c), increasing its surface area. It can be found that the alterations in surface morphology in Fig. 4c were the most apparent among the Fig. 4c–e, presenting the characterization results of ultrasonic extraction, SD extraction and maceration extraction treatments on the *C. wenyujin* samples, respectively, for 20 min. Furthermore, extending SD and maceration extraction times to 1 and 2 h, respectively, failed to bring similar serious morphological changes in Fig. 4f and g to that in Fig. 4c. Combined with the data in Table 3, we believed that the characterization changes (e.g. loose, damaged and rough) of surface morphology increased the extraction yields of the four compotents from *C. wenyujin*. Our results are agreement with those of prior researches indicating ultrasound could apparently change the surface morphology of raw samples because of the surface cavitation [35, 36]. Moreover, the “mechanoacoustic effects” is able to promote the availability of the phytomass through microjet erosion, cell wall disruption and mass transfer expansion in a heterogeneous mixture of phytomass and liquid, leading to an enhanced extraction efficiency [37]. In summary, ultrasonic extraction could produce cavitation and promote the expansion of the medicinal samples resulting in serious changes in surface morphology, which improve the specific surface area, extraction solvent penetration into herbal materials and release of intracellular soluble ingredients to solvent. Thus, ultrasonic extraction is suitable for extracting the four compotents from *C. wenyujin* with advantages of short extraction time and high efficiency.

### Antiproliferative activities

**Fig. 5:**
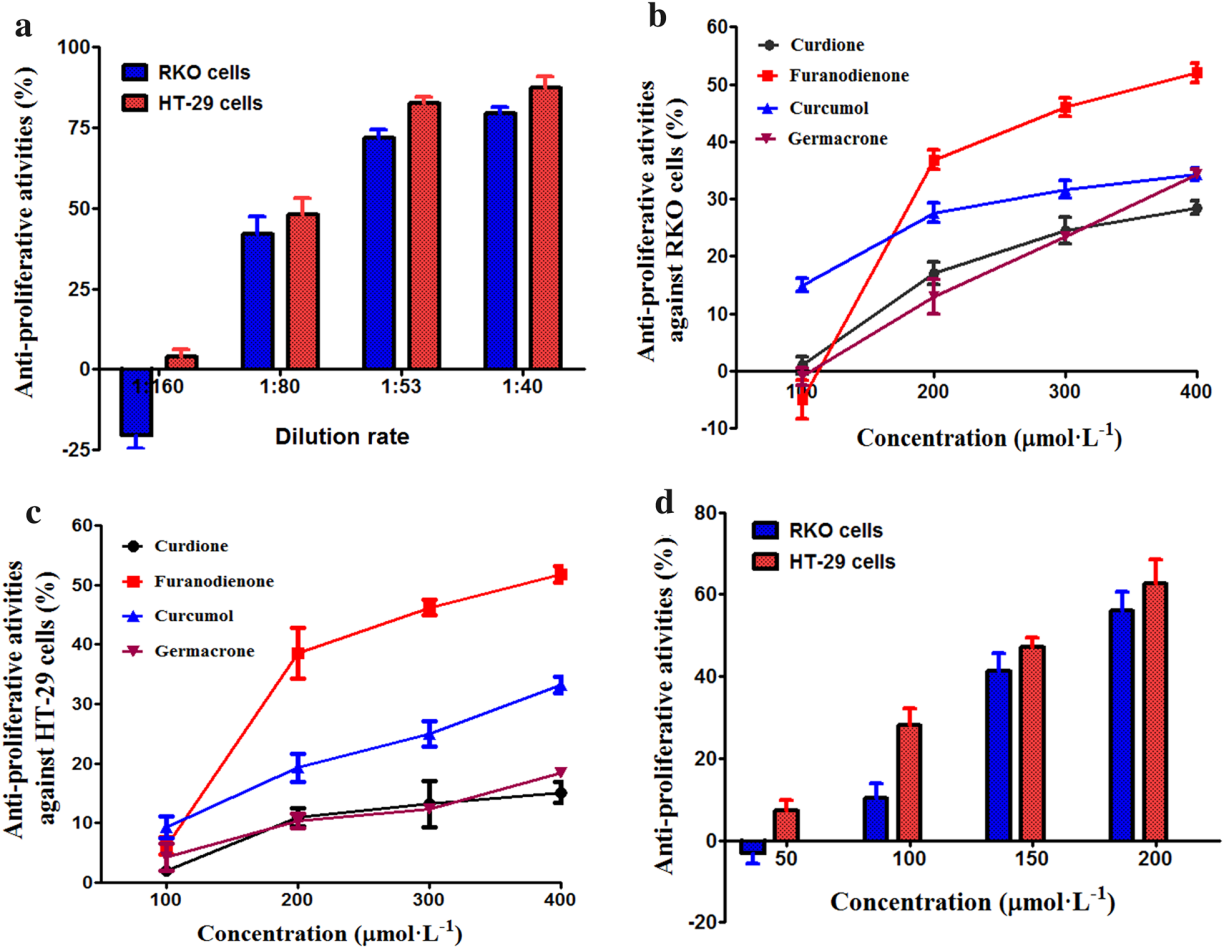
Antiproliferative activities in CRC cells **a** the effect of *Curcuma wenyujin* extract on RKO and HT-29 cells; **b** the effect of the four compounds on RKO cells; **c** the effect of the four compounds alone on HT-29 cells and **d** combined effect of these four compounds on RKO and HT-29 cells. In picture d, the concentration in horizontal coordinate refers to that of curdione

The evaluation of whether the *C. wenyujin* extract could effect the proliferation of RKO and HT-29 cells was performed using the CCK-8 assay. As displayed in Fig. 5a, the extract of *C. wenyujin* gained under the optimal ultrasonic extraction conditions reduced the growth of the two cells concentration-dependently at 1:80, 1:53 and 1:40 dilution rate after 48 h. The highest diluted extract (1:160 dilution) did not inhibit the growth of RKO cells, consistent to a previous research [38]. While, the antiproliferative rate against RKO cells was 79.5 % at 1:40 dilution. After RKO and HT-29 cells were treated with the extract for 48 h, the cell proliferation were observed with half inhibitory concentration (IC_50_) values of 1:67 and 1:76 dilution rates, respectively. Therefore, the extract of *C. wenyujin* exhibited remarkable antiproliferative potentials against the two cell lines for 48 h.

In order to ascertain which the compotent(s) in the extract could play a role in the the antiproliferative activity, these four main compotents of the extract were individually tested. Results, demonstrated in Fig. 5b and c, indicated that, all the four components showed significant growth inhibitory effects on the two cells except furanodienone on RKO cells at concentration 100 µmol L^−1^. Among these four bioactive compotents, furanodienone, whose content was the second highest in the *C. wenyujin* extract (Table 3), inhibited the growth of the two cell lines obviously, at concentration 200–400 µmol L^−1^, consistent to other studies [7, 39–41]. Wang et al. reported that curcumol was capable of inhibiting the cell viability of another two CRC cell lines in a concentration-dependent manner [42]. In this study, we further found that the inhibition rates of furanodienone against RKO and HT-29 cells were more than 50.0 % (52.0 and 51.7 %, respectively) at 400 µmol L^−1^, indicating strong antiproliferative potential.

The joint inhibitory functions of the four components on the two cells were also investigated at the concentration corresponding to that in Fig. 5a, as displayed in Fig. 5d. The mixed solution displayed concentration-dependent antiproliferative potentials against the two cells except against RKO cells at 50 µmol L^−1^. Besides, at concentration of 200 µmol L^−1^, the inhibition rates were 56.3 and 63.4 %, to RKO and HT-29 cells, respectively. In addition, the inhibitory actions of the mixed solution on HT-29 cells were higher than that on RKO cells, at the lower two concentrations (*p* < 0.05, Fig. 5d). This phenomenon may be explained that RKO cells were little less sensitive to low drug concentrations than HT-29 cells [43].

As compared Fig. 5a and d with Fig. 5b and c, it was obvious that the antiproliferative activities of single component against RKO and HT-29 cells were lower than those of the *C. wenyujin* extract or the mixture, at the same concentration. It may be related to the interactions among active components. For instance, the inhibitory potential of furanodiene on proliferation of breast cancer cells could be enhanced by germacrone [9]. Moreover, the active components in zedoary oil probably have a synergy on AGS cell growth [44]. Therefore, the antiproliferative activities of the *C. wenyujin* extract and the mixed solution against the two cell lines may be caused by the synergistic inhibition action of these components, which needs further investigation. Actually, synergistic action can exist in herbal medicine, decreasing active concentration of pure compound [38, 45]. As compared Fig. 5a with Fig. 5d, it can be seen that the proliferation inhibitory effects of the *C. wenyujin* extract on the two cell lines were slightly stronger than those of the mixture at the same concentration. A possibility for this result might be that other compotents existed in the total extract (Fig. 1) which could also be conducive to its overall antiproliferative activity, resulting in a series of complex combined effects.

In conclusion, the extract of *C. wenyujin* gained under the optimal ultrasonic extraction conditions demonstrated marked antiproliferative activities against RKO and HT-29 cells in vitro. The molecular mechanism of the antiproliferative activity needs to be further explored.

## Conclusions

This study was conducted to model and optimize the ultrasonic extraction conditions of extracting curdione, furanodienone, curcumol and germacrone from *C. wenyujin* by employing RSM and evaluate the inhibitory potential of the *C. wenyujin* extract on proliferation of RKO and HT-29 cells. Quadratic models for the four compounds content were derived with *R*^*2*^ in the range of 0.9435–0.9721. The simultaneous optimization of the multi-response system by DF indicated that the *D* of 97.1 % can be possible under the conditions: liquid–solid ratio, 8 mL g^−1^; ethanol concentration, 70 % and ultrasonic time, 20 min. Ultrasonic treatment effectively promoted the loose and rough morphology of *C. wenyujin* samples. Additionally, the *C. wenyujin* extract gained under the optimal ultrasonic extraction conditions exhibited remarkable antiproliferative activities against the two cell lines. In summary, the response surface methodology could been successfully employed to optimize the ultrasonic extraction of *C. wenyujin*, and the results demonstrates that the extract possesses a remarkable antiproliferative activity against colorectal cancer cells in vitro.

## Experimental

### Materials

*Curcuma wenyujin* Y.H. Chen et C. Ling, which grew in Zhejiang Province (China), was purchased from Shanghai General Hospital (China). The plant sample was ground into powder using a cyclone mill, and the powder was sieved through a 60 mesh sieve for ultrasonic extraction. HPLC-grade methanol and acetonitrile were brought from TEDIA (Ohio, USA). Ethanol, ether and ethyl acetate were analytically pure and obtained from Sanjie Chemical Co., Suzhou, China. Pure water was gained from a Millipore Milli Q-Plus system (Millipore, Bedford, MA). Curcumol, curdione and germacrone were obtained from Standard Bio-Technology Co., Ltd, Shanghai, China. Furanodienone was purchased from Yuanye Bio-Technology Co., Ltd, Shanghai, China. Dulbecco’s modified Eagle’s medium (DMEM), antibiotics (penicillin–streptomycin) and phosphate-buffered saline (PBS) were obtained from Jinuo Biotechnology (Hangzhou, China). Fetal bovine serum (FBS) was supplied by Gibco (CA, USA). CCK-8 kit was obtained from Dojindo Laboratories (Tokyo, Japan). Dimethyl sulfoxide (DMSO) was from Sigma (MO, USA).

### Methods

#### High-performance liquid chromatography

Agilent Series 1100 liquid chromatography (Agilent Technologies, USA) with a Zorbax C18 column (4.6 × 150 mm, 5 µm) was adopted for HPLC analysis. The elution system was: acetonitrile, solvent A; water, solvent B. The gradient elution conditions applied were: 0–10 min, linear gradient 50–60 % A and 10–20 min, linear gradient 60–80 % A. The column temperature was 25 °C. The injection volume was 20 µL, and the flow-rate was 1 mL min^−1^. The peaks were detected at 210 nm.

#### Single factor tests

Taking previous researches and the constraints of experimental equipment into consideration [46], ultrasonic extraction was performed using an ultrasonic cleaning bath at 250 W and 25 kHz. The influences of five parameters, namely the type of solvent, solvent concentration, liquid–solid ratio, ultrasonic time and extraction temperature, on the total extraction yields of curdione, furanodienone, curcumol and germacrone from *C. wenyujin* were examined by single factor tests. Firstly, the extraction abilities of methanol, ethanol, ether and ethyl acetate were examined. After ethanol was chosen as the suitable extraction solvent, the ethanol concentration was investigated at 30 °C with 10.0 g samples and 80 mL ethanol solutions at concentrations of 40, 60, 70, 80 and 100 % for 10 min. After 70 % ethanol solution was chosen as the optimum extraction solvent, 10.0 g samples were sonicated with different liquid–solid ratios (4, 6, 8 and 10 mL g^−1^) for 10 min at 30 °C. Then, the ultrasonic time (3, 5, 10, 15 and 20 min) was investigated with 80 mL extraction solvent at 30 °C. Finally, to evaluate the influence of temperature, 10.0 g samples with 80 mL extraction solvent were sonicated 15 min at 20, 30, 40 and 50 °C, respectively.

#### Ultrasonic extraction

Ultrasonic extraction was carried out for extracting the main four compotents from *C. wenyujin* sample. Firstly, 10.00 g *C. wenyujin* sample and a certain volume of solvent were placed into a 100 mL flask and sonicated at a fixed temperature for a given time. After extraction, the extract was centrifugated at 6000 rpm for 10 min. Subsequently, the supernatant extracted using methanol, ethanol or ethanol solution was poured into a 100 mL volumetric flask which was then filled to the mark with extraction solvent. Meanwhile, the supernatants extracted using other two solvents were evaporated and then dissolved with methanol. Lastly, each extracted solution was filtered with a 0.45 µm econofilter for determination analysis by HPLC.

#### Central composite design

On the basis of single factor tests, a three-variable, five-level CCD with 17 runs was built (Table 1) [47]. The ultrasonic treatments were conducted in random to minimize systematic errors [17]. Design-Expert™ version 8.5 software (Stat-Ease Inc., Minneapolis, MN, USA) was adopted to analyze the data and estimate the regression equation coefficients [48]. The form of quadratic response model was as follows:

5 $$ Y_{f} = \beta_{0} + \sum\limits_{i = 1}^{3} {\beta_{i} X_{i} + \sum\limits_{i = 1}^{3} {\beta_{ii} X_{i}^{2} + \sum\limits_{i = 1}^{2} {\sum\limits_{j = i + 1}^{3} {\beta_{ij} X_{i} X_{j} } } } } $$

where *β*_*0*_; *β*_*i*_; *β*_*ii*_ and *β*_*ij*_ are the coefficients for the response surface model. *X*_*i*_ and *X*_*j*_ are the independent variables. *Y*_*f*_ is the measured response variable.

#### Desirability function

A DF approach was employed to optimize the four responses simultaneously. The principle is to transform each predicted response to a dimensionless desirability (*d*_*i*_) between 0 and 1, and combine their geometric average of the *d*_*i*_ values into *D*. The equation was as follows [17]:

6 $$ D = \text{(}d_{1} \times d_{2} \times d_{3} \times \cdot \cdot \cdot \times d_{n} \text{)}^{1/n} = \left( {\prod\limits_{i = 1}^{n} {d_{i} }} \right)^{1/n} $$

where *n* indicates the number of characteristics.

The bound of each response and parameter was defined by the results in Table 1, and the “Goal” field for each response was set to the “maximum” to obtain the maximum *D*.

#### Comparison and field emission scanning electron microscope

In order to compare the extraction ability of the ultrasonic extraction technique to that of the classical extraction methods and investigate the mechanism of ultrasonic extraction, ultrasonic extraction, SD extraction and maceration extraction were all carried out with a same liquid–solid ratio (8 mL g^−1^). After centrifugation, HPLC was employed for determination the extraction yields of the four compounds in the *C. wenyujin* extract. Meanwhile, to protect the original structures of these precipitates from damage, the dry process was performed on a vacuum freezerdrye (FreeZone Stoppering Tray Dryer, Labconco) [49]. Micrographs about the surface morphologies of these samples were obtained with FESEM.

#### Cell culture and CCK-8 assay

CRC RKO and HT-29 cells provided by the Institute of Clinical Translational Research, Shanghai General Hospital (Shanghai, China) were incubated in DMEM with 1 % antibiotics and 10 % FBS at 37 °C and 5 % CO_2_.

Firstly, the raw *C. wenyujin* extract obtained under the optimized ultrasonic extraction conditions was concentrated for 10 times to eliminate the influence of ethanol on cytoactive by vacuum concentration method. The concentrated *C. wenyujin* extract was then diluted with DMEM, antibiotics and FBS to 1:160, 1:80, 1:53 and 1:40 solutions. Meanwhile, these four pure ingredients were dissolved by DMSO to prepare stock solutions and then diluted as needed. Based on the concentration proportion of these main four compounds in the extract of *C. wenyujin*, the mixed solutions were prepared. The concentration of curdione in this mixture was used to mark that of the mixed solution.

The antiproliferative activities of the *C. wenyujin* extract against the two kinds of tumor cells were tested by a CCK-8 kit. Briefly, the two cells were counted and seeded into 96-well plates with a density of 5 × 10^3^ and 8 × 10^3^ cells per well, respectively, and allowed to adhere to the plates overnight. Subsequently, the cells were treated with a range of dilution ratios of *C. wenyujin* extract for 48 h. Lastly, the absorbance was monitored at 450 nm using Microplate reader (BIO-RAD, CA, USA). Similarly, the separate or joint effects of the main four compotents in *C. wenyujin* extract on the proliferation of the two cells were also examined.

#### Statistical analysis

All analyses were carried out at three times. The CCD results were analyzed by Design-expert version 8.5 software. The comparison of the actual and the predictive value of these four models was performed by the SRD analyses. IBM SPSS 20.0 software (SPSS Inc., Chicago, IL, USA) was adopted to perform the ANOVA for the extraction yields of different extraction methods and calculate IC_50_. In the present study, *p* < 0.05 was considered as statistically significant.

## Additional files

10.1186/s13065-016-0177-9 Analytical performance of these four investigated compounds in *Curcuma wenyujin* by the HPLC method.
10.1186/s13065-016-0177-9 Effects of five factors on the total extraction yield of curdione, furanodienone, curcumol and germacrone from *Curcuma wenyujin*. (a) type of solvent; (b) ethanol concentration; (c) liquid–solid ratio; (d) ultrasonic time and (e) temperature.
10.1186/s13065-016-0177-9 Predicted responses versus actual responses. (a) curdione; (b) furanodienone; (c) curcumol; and (d) germacrone.
10.1186/s13065-016-0177-9 Bar graph showing individual desirability values (*d*_*i*_) of various objective responses and the maximum combined desirability of 0.971 for the optimization of ultrasonic extraction conditions for extraction of curdione, furanodienone, curcumol and germacrone from *Curcuma wenyujin.*

## Abbreviations

SFE: supercritical fluid extraction
RSM: response surface methodology
CCD: central composite design
DF: desirability function
FESEM: field emission scanning electron microscope
HPLC: high performance liquid chromatography
SD: steam distillation
RSD: relative standard deviations
CCK-8: cell counting kit-8
CRC: colorectal cancer
ANOVA: analysis of variance
*D*: combined desirability
IC_50_: half maximal inhibitory concentration
SRD: sum of ranking differences
