# Supplementary material for: Optimization and in vitro antiproliferation of Curcuma wenyujin’s active extracts by ultrasonication and response surface methodology
Source: Chem Cent J. 2016 May 16;10:32. doi: 10.1186/s13065-016-0177-9 (PMC4868111; doi:10.1186/s13065-016-0177-9)
Supplement: Supplementary file 1 — 10.1186/s13065-016-0177-9 Analytical performance of these four investigated compounds in Curcuma wenyujin by the HPLC method. [file 13065_2016_177_MOESM1_ESM.docx]

**Table S1** Analytical performance of these four investigated compounds in *Curcuma wenyujin* by the HPLC method.

| **Analytes** | **Regressive equation**  **(*X*: mg·mL^−1^)** | ***r^2^*** | **Test range (mg·mL^−1^)** | **LOD ^a^ (ng)** | **LOQ ^b^ (ng)** | **Recovery (%)** | **RSD ^c^ (%)** |
| --- | --- | --- | --- | --- | --- | --- | --- |
| Curdione | *Y* = 16305*X* + 28.80 | 0.9999 | 0.016-1.050 | 8.42 | 28.48 | 101.96 | 3.69 |
| Furanodienone | *Y* = 61202*X* + 345.22 | 0.9994 | 0.012-0.373 | 7.77 | 23.31 | 101.03 | 4.82 |
| Curcumol | *Y* = 40914*X* − 59.26 | 0.9998 | 0.004-0.254 | 7.94 | 29.38 | 104.28 | 4.64 |
| Germacrone | *Y* = 72213*X* + 24.06 | 0.9999 | 0.003-0.194 | 9.70 | 24.25 | 97.91 | 3.90 |

^a^ LOD means limit of detection; ^b^ LOQ means limit of quantification;

^c^ RSD means relative standard deviation, RSD (%) = 100×SD/mean.
