# Supplementary figures and images for: Optimization and in vitro antiproliferation of Curcuma wenyujin’s active extracts by ultrasonication and response surface methodology
Source: Chem Cent J. 2016 May 16;10:32. doi: 10.1186/s13065-016-0177-9 (PMC4868111; doi:10.1186/s13065-016-0177-9)

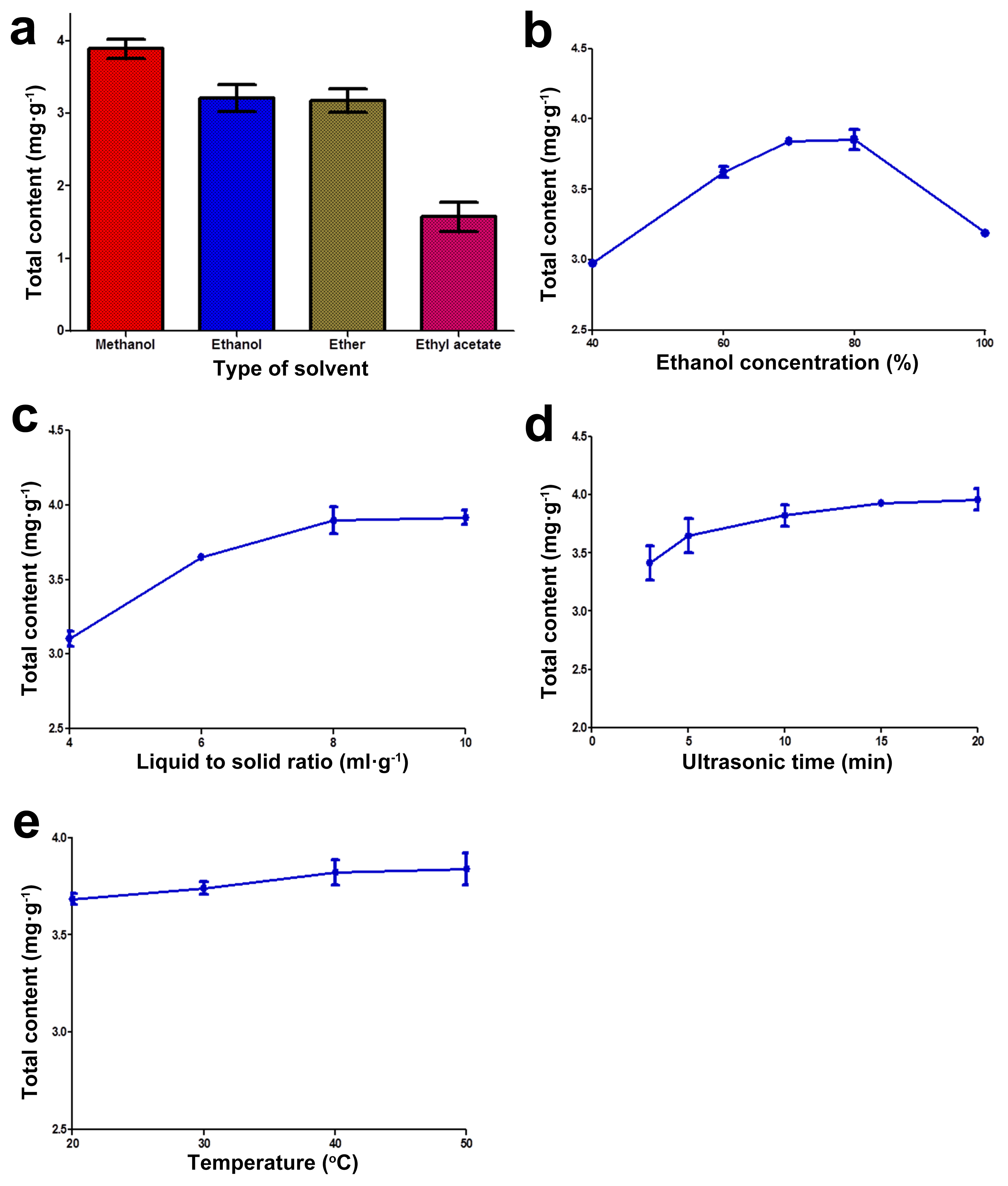

Supplement: Supplementary file 2 — 10.1186/s13065-016-0177-9 Effects of five factors on the total extraction yield of curdione, furanodienone, curcumol and germacrone from Curcuma wenyujin. (a) type of solvent; (b) ethanol concentration; (c) liquid–solid ratio; (d) ultrasonic time and (e) temperature. [file 13065_2016_177_MOESM2_ESM.tif]

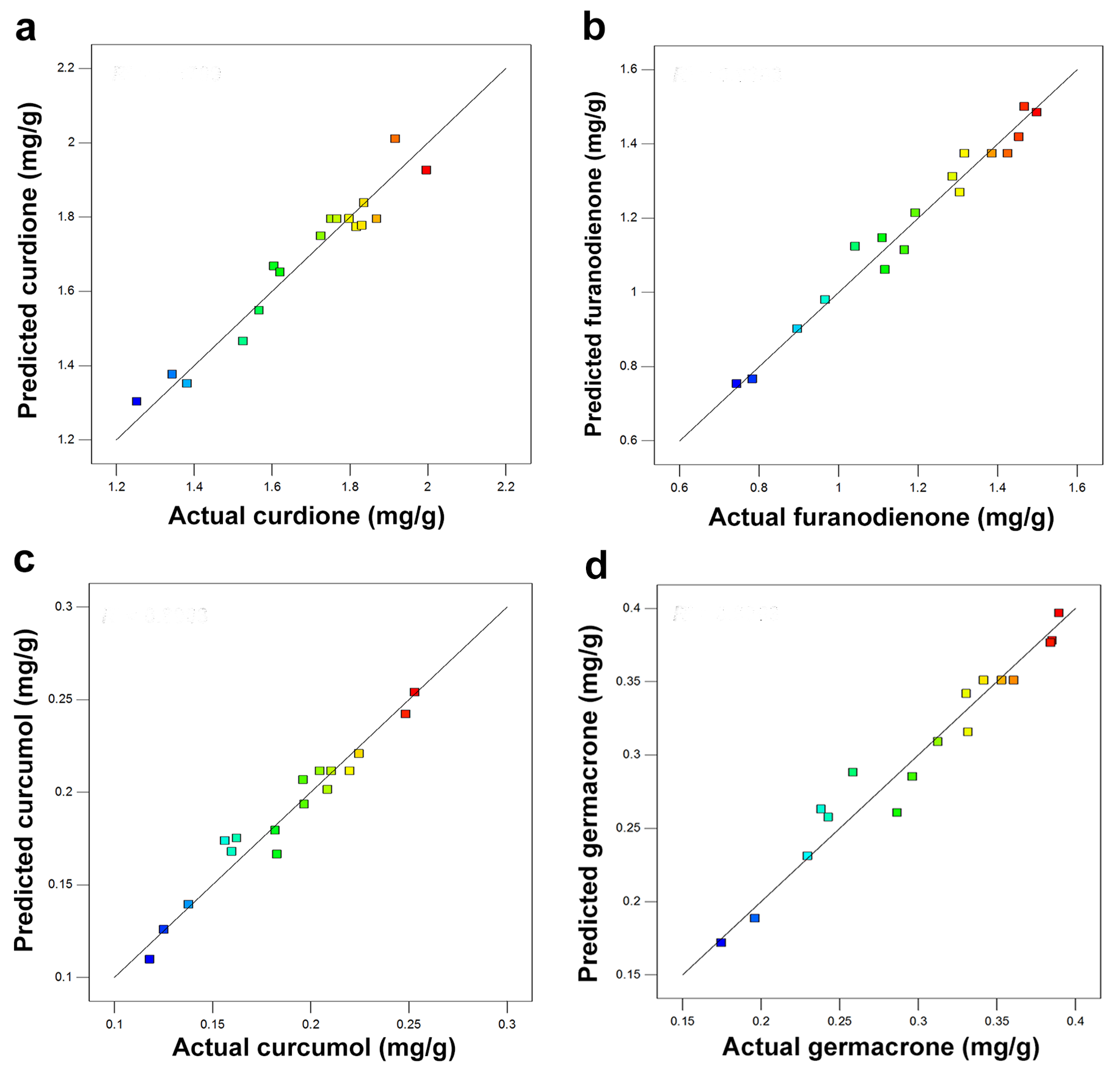

Supplement: Supplementary file 3 — 10.1186/s13065-016-0177-9 Predicted responses versus actual responses. (a) curdione; (b) furanodienone; (c) curcumol; and (d) germacrone. [file 13065_2016_177_MOESM3_ESM.tif]

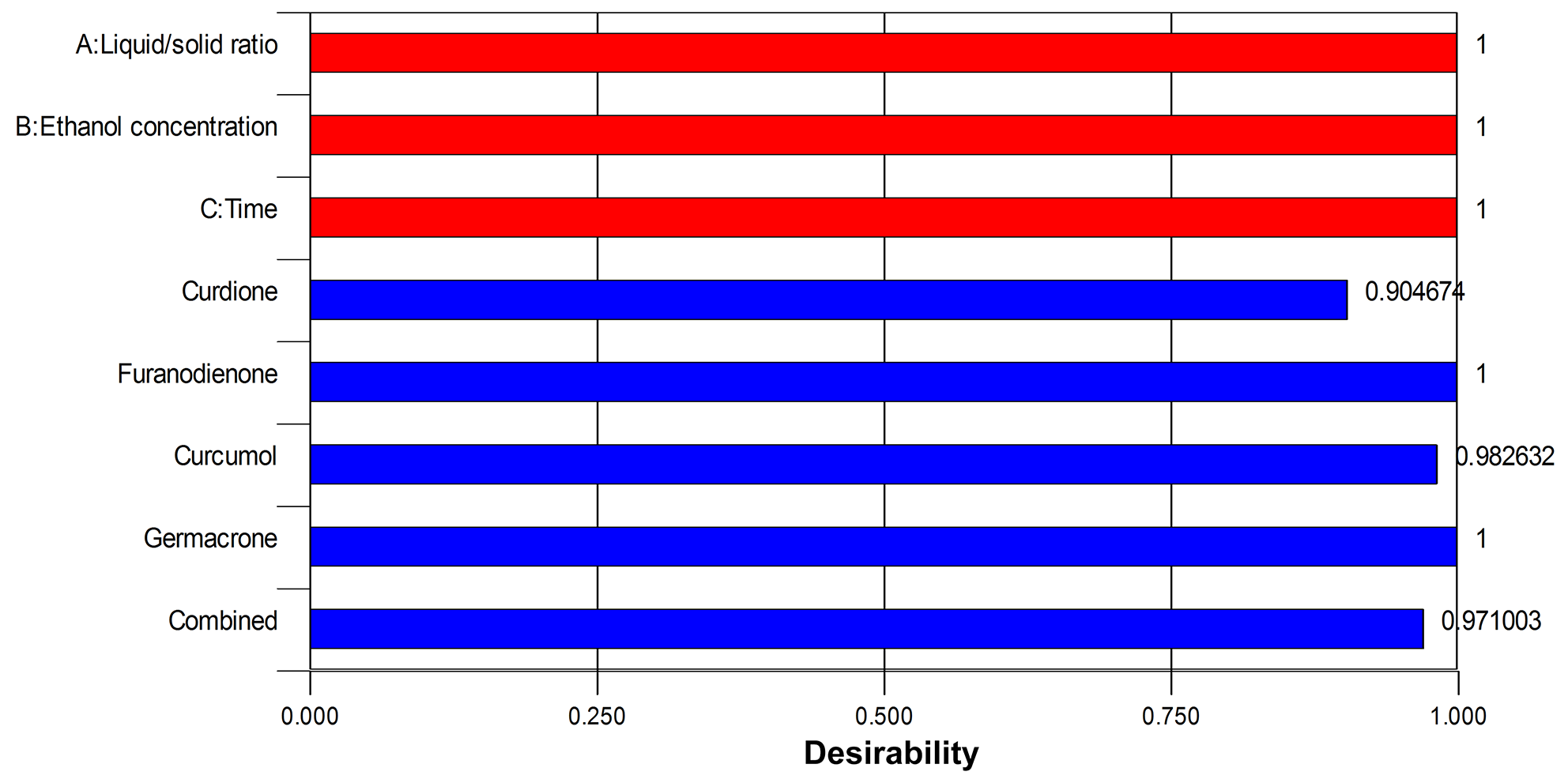

Supplement: Supplementary file 4 — 10.1186/s13065-016-0177-9 Bar graph showing individual desirability values (d i) of various objective responses and the maximum combined desirability of 0.971 for the optimization of ultrasonic extraction conditions for extraction of curdione, furanodienone, curcumol and germacrone from Curcuma wenyujin. [file 13065_2016_177_MOESM4_ESM.tif]
